# A Novel Medical Freehand Sketch 3D Model Retrieval Method by Dimensionality Reduction and Feature Vector Transformation

**DOI:** 10.1155/2016/4738391

**Published:** 2016-05-17

**Authors:** Zhang Jing, Kang Bao Sheng

**Affiliations:** Northwestern University, Xi'an, Shanxi 710127, China

## Abstract

To assist physicians to quickly find the required 3D model from the mass medical model, we propose a novel retrieval method, called DRFVT, which combines the characteristics of dimensionality reduction (DR) and feature vector transformation (FVT) method. The DR method reduces the dimensionality of feature vector; only the top *M* low frequency Discrete Fourier Transform coefficients are retained. The FVT method does the transformation of the original feature vector and generates a new feature vector to solve the problem of noise sensitivity. The experiment results demonstrate that the DRFVT method achieves more effective and efficient retrieval results than other proposed methods.

## 1. Introduction

The medical 3D model retrieval becomes a hot research topic due to the rapid development in clinic and teaching. The medical 3D model can not only show doctors the anatomical structure of a particular part inside the human body, but also reveal the function of human organs to a certain degree [1]. The current diagnostic techniques, especially in the cases which cannot immediately get the diagnosis, need the auxiliary diagnosis by previous similar cases. At this time, 3D model retrieval technology is applied to obtain the similar 3D model from the database. In the face of vast numbers of medical 3D models, how to find the required 3D model rapidly and extract the valuable information accurately has become an urgent issue [2].

The most popular way for retrieving 3D models is example-based paradigm [3], where the user provides an existing 3D model as query input and the retrieval method can return similar 3D models from the database. However, it is difficult for a user to have an appropriate example 3D model at hand. An alternative way is to use a 2D sketch as a query where users can describe a target 3D model by quickly drawing it. But a 2D sketch is merely a coarse and simple representation which only contains partial information of an original 3D model. Hence, it is more challenging to realize freehand retrieval [4] than example-based retrieval.

For the freehand sketch 3D model retrieval how to create efficient feature descriptors [5] is the most important part. Several freehand sketch 3D model retrieval methods have been proposed recently. Funkhouser et al. [6], Chen et al. [7], and Li et al. [8] extracted feature descriptors from the outline to describe the query sketch and 2D views of 3D models, such as the spherical harmonics descriptor, light field descriptor, and shape context features. Recently, Wang et al. [9] provided a sketch-based retrieval approach by utilizing both global feature and local feature. In the practical applications, we find that these features are not effective and efficient in evaluating the relevance between the query sketch and 3D models. There exist two main disadvantages for the current freehand sketch 3D model retrieval methods. Firstly, the traditional Fourier Transform [10] only considers describing the model and the argument of the coefficient. Compactness [11] and simplicity of feature descriptors are necessary for minimizing the storage overhead and the computing time. Therefore, it is necessary to describe the outline by a limited number of coefficients in the frequency domain. Secondly, a freehand sketch is a simple line drawing which has a high level of abstraction, inherent ambiguity, and unavoidable noise [12]. For effective retrieval, the feature descriptors should be robust to noise and invariant to transformations.

To tackle these problems, this paper proposes a novel freehand sketch 3D medical model retrieval method, which combines the characteristics of dimensionality reduction (DR) and feature vector transformation (FVT) method, called DRFVT. The DR method reduces the dimensionality of feature vector; only the top *M* low frequency Discrete Fourier Transform (DFT) coefficients are retained. These *M* coefficients are able to provide all of the abovementioned requirements and obtain good efficiency in retrieval [13]. The FVT method does the transformation of the original feature vector and generates a new feature vector to solve the problem of noise sensitivity. On this basis, the similarity between the sketch and the 3D model is calculated [14]. To evaluate our method, we test our method on the public standard data set and also compared with other leading 3D model retrieval approaches [15]. The experiments demonstrate that the DRFVT method is significantly better than any other retrieval techniques.

## 2. Our Method

**Figure 1:**
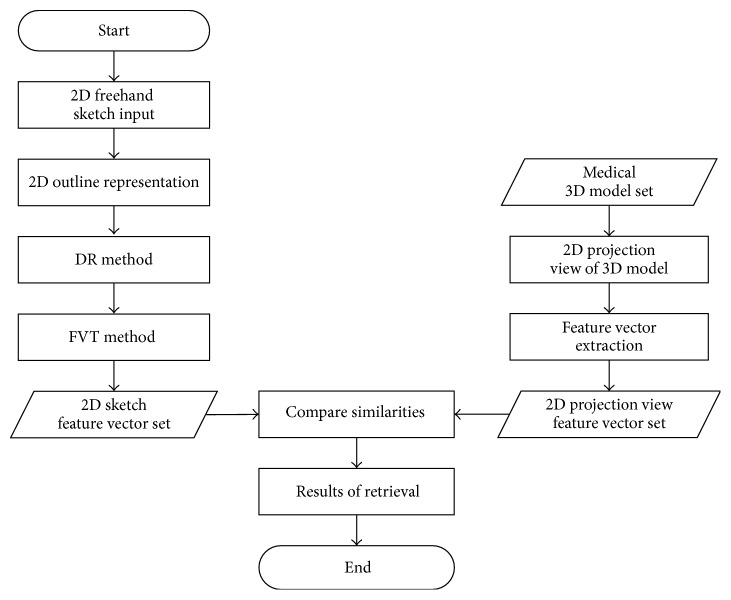
The framework of the medical freehand sketch 3D model retrieval method.

This paper researches on how to get user's retrieval intention and the feature extraction of 2D freehand sketch. Users express their retrieval intention freely by freehand sketch. In order to realize the 3D model retrieval, this paper calculates the similarity between 2D sketches feature vector and 3D model projection view feature vector. The framework of the medical freehand sketch 3D model retrieval method is proposed, as shown in Figure 1 [16, 17].

The 2D freehand sketch framework contains five modules, respectively, 2D freehand sketch input module, 2D outline representation module, DR method module, FVT method module, and 2D sketch feature vector set module. The 2D freehand sketch input module would get user's retrieval intention with hand drawing or mouse drawing. The 2D outline representation module extracts the outline of sketch based on the eight-direction adaptive tracking algorithm [18]. The DR method module reduces the dimensionality of feature vector; only the top *M* low frequency Discrete Fourier Transform coefficients are retained. The FVT method module does the transformation of the original feature vector and generates a new feature vector to solve the problem of noise sensitivity. The 2D sketch feature vector set module contains the feature vector set generated by the DR method and the FVT method.

The medical 3D model framework contains four modules, respectively, medical 3D model set module, 2D projection view of 3D model module, feature vector extraction module, and 2D projection view feature vector set module. The medical 3D model set module contains 300 3D model files of medical organs from the Princeton Shape Benchmark [19]. The 2D projection view of 3D model module chooses and generates 2D projection views of each 3D model. The feature vector extraction module creates feature descriptors which describe the structural information and recognize interior content from 2D projection views of 3D model. The 2D projection view feature vector set module contains the feature vector set of 2D projection views of medical 3D model, which we use to compare with the 2D sketch feature vector set.

### 2.1. Feature Extraction of 2D Sketch

In the general sketch retrieval method, the sketches are composed of element figures, and the relationship between the element's figures is complex. For the 3D model retrieval method, the sketches are not expressed by simple element figures, because the outline of the sketch is complicated and has abundant feature information; it cannot be expressed by simple element figures. Therefore our method firstly extracts the outline of the sketch based on the eight-direction adaptive tracking algorithm and parameterizes the outline to obtain a complex discrete-time periodic signal whose period *N* equals the number of points. Secondly, we adopt the DR method to reduce the dimensionality of feature vector, which obtains good efficiency in retrieval. Finally, we propose that the FVT method does the transformation of the original feature vector, which uses the new feature vector to solve the sensitive noise problem [20–22].

#### 2.1.1. 2D Outline Representation

**Figure 2:**
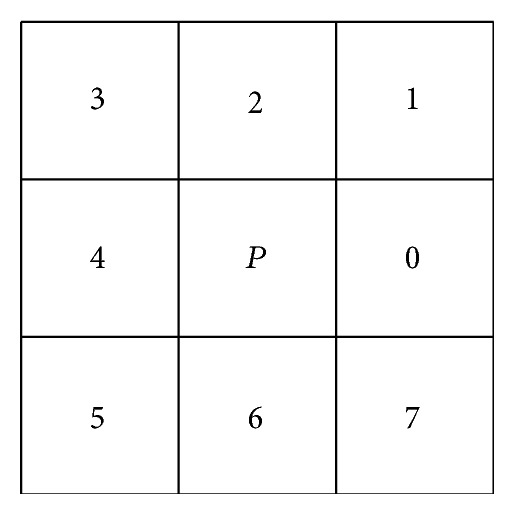
The point *P* eight directions.

For the sketches submitted by users, this paper extracts the outline of the sketch based on the eight-direction adaptive tracking algorithm. Let *I*(*x*, *y*) represent the original image [23] and *C*(*x*, *y*) represent the outline binary image. As shown in Figure 2, we search the outline in the 3 × 3 region with point *P* as centre counterclockwise.

This algorithm selects the first point of *I*(*x*, *y*) as *first*_*p*. If there are no other points in direction 2,3, 4 of *first*_*p* counterclockwise, we save *first*_*p* into the outline binary image *C*(*x*, *y*). The algorithm is described as follows. 

*Algorithm  1*

*Step  1* (initialization). Set the outline binary image *C*(*x*, *y*) to be null. The direction array DI is assigned by DI = {0,1, 2,3, 4,5, 6,7}. Let *d* represent the current direction, and *d* = 2. 

*Step  2*. Scan the original image *I*(*x*, *y*) line by line in order, from top to bottom and left to right. We obtain the starting point *first*_*p* of the outline and initialize the current point *now*_*p* with *first*_*p*. 

*Step  3*. Add *now*_*p* to the outline binary image *C*(*x*, *y*). *next*_*p* represents the next adjacent point of *now*_*p*. Search *next*_*p* according to the direction array order DI[*d*], DI[*d* + 1],…, DI[*d* + 7]. We assume the *next*_*p* direction is DI[*i*]; the new search direction is *d* = (DI[*i*] + 4 + 1)mod⁡8. Then assign *now*_*p* by *next*_*p* again. 

*Step  4*. Judge whether *now*_*p* coincides with the starting point *first*_*p*; if yes then exit; otherwise returns Step 3.

#### 2.1.2. The DR Method Based on Fourier Transform

(*1) Method Description*. The outline of the sketch is closed and cyclical. We can use Fourier Transform to describe its characteristics. First of all, we consider the outline of an object as a discrete-time complex periodical signal *z* = 〈*z*_0_, *z*_1_,…, *z*_*N*−1_〉 and define $z_{l}=x_{l}+iy_{l}\;\;(i=\sqrt{-1})$, where *x*_*l*_ and *y*_*l*_ are the real coordinate values of the *l*th (*l* = 0,…, *N* − 1) sampled point. The signal *z* is then mapped to the frequency domain by the DFT [24],

(1) Zm=∑l=0N−1zle−i2πlm/N=Rmeiθmm=−N2,…,−1,0,1,…,N2−1,

where *R*_*m*_ and *θ*_*m*_ are the module and the argument of the *m*th DFT coefficient, respectively. 

(*2) Invariance Requirement*. In order to guarantee the translation, scaling, rotation, and starting point invariance, we have to modify the DFT coefficients accordingly. When a change is in the position, size, orientation of the object, or the initial point used to parameterize the outline, the DFT coefficients are modified [25]. In Table 1, we show how normalized DFT coefficients ${\overset{⌢}{Z}}_{m}={\overset{⌢}{R}}_{m}e^{i{\overset{⌢}{\theta }}_{m}}$ satisfying the invariance requirements can be obtained.

If one wants to achieve rotation and starting point invariance, the two corresponding modifications have to be integrated, leading to

(2) $$\begin{array}{c} {\overset{⌢}{\theta }}_{m}=\theta_{m}-\frac{\theta_{-1}+\theta_{1}}{2}+m\frac{\theta_{-1}-\theta_{1}}{2}. \end{array}$$

For rotation and starting point invariance, the derivation is as follows [26–28].

We consider the original outline signals *z* and *z*′, where *z*′ is obtained from *z* by rotating each point counterclockwise by a constant factor *θ*_0_ and by shifting the starting point by *l*_0_ positions, *z*_*l*_′ = *z*_*l*−*l*_0__ *e* ^*iθ*_0_^. The corresponding DFT coefficients are

(3) $$\begin{array}{c} Z_{m}^{\prime }=Z_{m}e^{i\theta_{0}}e^{-i\left(2\pi l_{0}m/N\right)}=R_{m}e^{i\left(\theta_{m}+\theta_{0}-2\pi l_{0}m/N\right)}. \end{array}$$

Thus, *θ*_*m*_′ can be expressed as

(4) $$\begin{array}{c} \theta_{m}^{\prime }=\theta_{m}+\theta_{0}-\frac{2\pi l_{0}m}{N}. \end{array}$$

In particular, when *m* is 1 or −1, *θ*_1_′ and *θ*_−1_′ are

(5) θ1′=θ1+θ0−2πl0N,θ−1′=θ−1+θ0+2πl0N.

By referring to Table 1, it is obtained that

(6) θ⌢m′θm′−θ−1′+θ1′2+mθ−1′−θ1′2=θm+θ0−θ−1+θ12−2πl0mN−θ0+mθ−1−θ12+2πl0mN=θm−θ−1+θ12+mθ−1−θ12=θ⌢m.

By simply performing the Inverse DFT on normalized DFT coefficients, we obtain a modified normalized signal *z*′ which satisfies all the requested invariance

(7) $$\begin{array}{c} {\overset{⌢}{z}}_{l}=\frac{1}{M}\overset{M/2-1}{\underset{m=-M/2}{\sum }}{\overset{⌢}{Z}}_{m}e^{i\left(2\pi lm/M\right)}\;\left(l=0,1,\ldots ,M-1\right). \end{array}$$

(*3) Dimensionality Selection*. In order to concentrate the outline information of the sketch, we use only low frequency DFT coefficients. In particular, we keep only the *M*  (*M* ≪ *N*) coefficients whose frequency is closer to 0. The DR method uses the Bartolini et al. [29] spectral characteristics *E*(*M*) to determine the value of *M*. The energy of the signal retained by the *M* coefficients is defined as

(8) $$\begin{array}{c} E\left(\;M\;\right)=\overset{m=M/2-1}{\underset{m=-M/2,m\neq 0}{\sum }}{\left|\;Z_{m}\;\right|}^{2}. \end{array}$$

The choice of a suitable value for *M* has to trade off the accuracy in representing the original signal with the compactness and the extraction efficiency.

#### 2.1.3. The FVT Method

The feature vector extraction algorithm is as follows.

Let the centroid be (*x*_0_, *y*_0_); the distance *r*_*t*_ from the outline point to the centroid is shown as below:

(9) $$\begin{array}{c} r_{t}=\sqrt{{\left(x_{t}-x_{0}\right)}^{2}+{\left(y_{t}-y_{0}\right)}^{2}}. \end{array}$$

Then the distance from each of *M* outline points to the centroid (*x*_0_, *y*_0_) generates the *M* dimension feature vector *R* = (*r*_1_, *r*_2_,…, *r*_*M*_).

The traditional Fourier Transform has certain sensitivity to noise, and the noise sensitivity problems will affect the similarity accuracy of feature vector. Therefore, this paper proposes the FVT method, which solves the issue of noise sensitivity. For the feature vector *R* = (*r*_1_, *r*_2_,…, *r*_*M*_), set *r*_*k*_ = min⁡(*r*_1_, *r*_2_,…, *r*_*M*_); then generate the new feature vector *R*′ = (*r*_*k*_, *r*_*k*+1_,…, *r*_*M*_, *r*_1_,…, *r*_*k*−1_).

### 2.2. Similarity Comparison

From the user input retrieval intention, retrieving the user's satisfactory models requires comparing the feature vector distances between the input sketch and the 3D model. The similarity of models is determined by the differences between feature vectors [30–34].

In order to realize the medical freehand sketch 3D model retrieval, this paper compares the similarities of the medical 3D model based on Euclidean distance [35], where *X*, *Y* represent the feature vector of 2D sketch and 2D projection views of the 3D model, respectively:

(10) $$\begin{array}{c} D\left(\;X,Y\;\right)=\sqrt{\overset{n}{\underset{i=1}{\sum }}{\left(x_{i}-y_{i}\right)}^{2}}. \end{array}$$

## 3. Experiments and Results

We implement the medical 3D model retrieval method in C++ under Windows. The system consists of a computer with an Intel Xeon CPU E5520@2.27 GHz and 12.0 GB of RAM. On average, each 3D model takes 6.1 seconds to extract features; before the sketch retrieval we can extract and store the 2D projection view feature vector. Moreover the 2D freehand sketch takes 0.06 seconds to extract features. The freehand sketch retrieval takes 0.1 seconds in the medical 3D model retrieval system.

For performance evaluation, we select 300 3D model files of medical organs from the Princeton Shape Benchmark [18] (http://shape.cs.princeton.edu/benchmark/); each 3D model is manually classified based on 6 categories (“Body” (130 images), “Heart” (30), “Lung” (25), “Ovary” (13), “Kidney” (22), and “Liver” (18)). The 3D models do not belong to any category and were assigned to a default class (62).  Table 2 shows some 3D models in the data set, along with their category.

**Figure 3:**
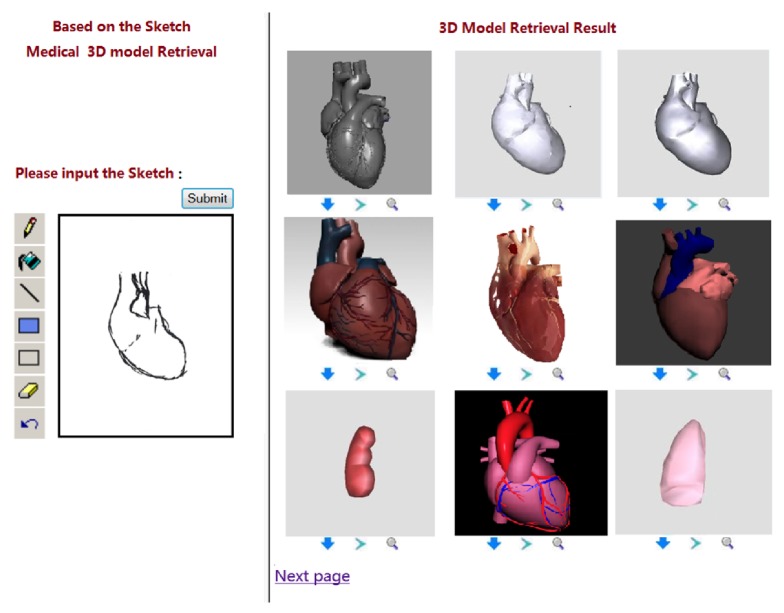
The medical freehand sketch 3D model retrieval system.

In this paper, we provide users' interface with hand drawing or mouse drawing, which is convenient for users to draw and modify the freehand sketches. As shown in Figure 3, the left is the tablet which is used for hand drawing or mouse drawing, and the right shows 3D model retrieval results.

### 3.1. The 2D Sketch Feature Extraction Experiments

#### 3.1.1. Extract the Outline of Sketch

**Figure 4:**
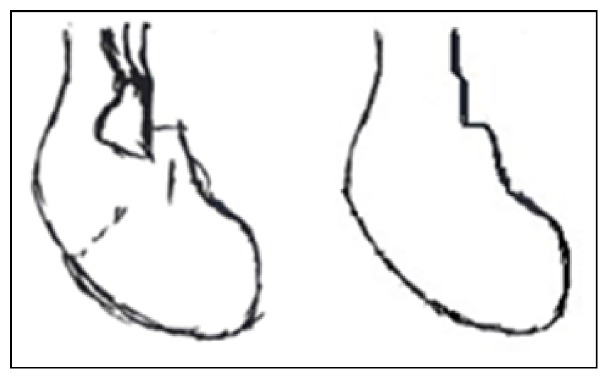
The effect of the outline extraction.

This paper extracts the outline of sketch based on the eight-direction adaptive tracking algorithm. It is assumed that when the sketch is drawn, the mouse point sequence is stored in *I*(*x*, *y*). After that *I*(*x*, *y*) is applied to generate the outline binary image *C*(*x*, *y*); Figure 4 shows a freehand sketch and the outline extraction.

#### 3.1.2. The DR Experiments

According to the above process, we use the eight-direction adaptive tracking algorithm to parameterize *N* points outline. The DR method reduces the dimensionality of feature vector; only the top *M* low frequency DFT coefficients are retained. We consider the spectral characteristics *E*(*M*) of the data set to determine the value of *M*.

**Figure 5:**
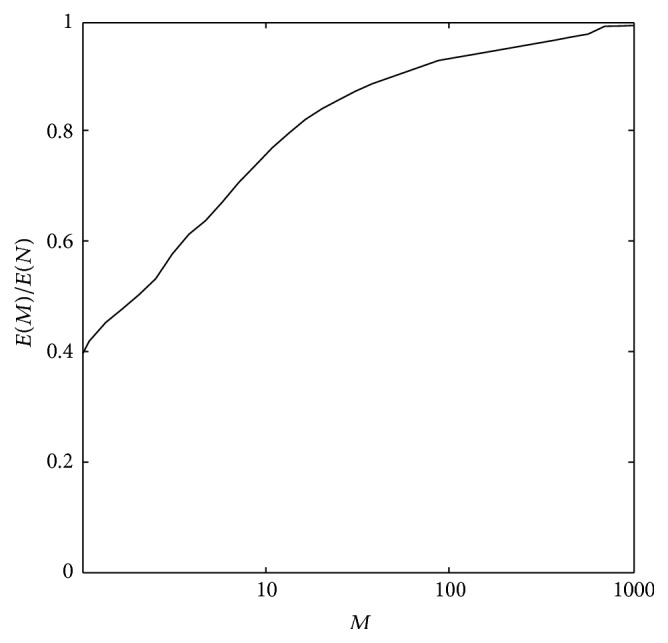
The value of *E*(*M*)/*E*(*N*) for the heart 3D model.

As an example, Figure 5 plots the value of *E*(*M*)/*E*(*N*) for the heart 3D model set. From the graph, when choosing *M* ∈ [16,64], the retained energy varies from 75 percent to 91 percent of the total. On the other hand, when obtaining *E*(*M*)/*E*(*N*) = 95%, *M* should be equal to 512. The *M* value will represent 512 outline points and then construct 512 vertices of graph, which is not obviously appropriate. Therefore choose the smaller *M*, such as *M* = 32; *M* is a power of 2; we can easily use the Fast Fourier Transform.

**Figure 6:**
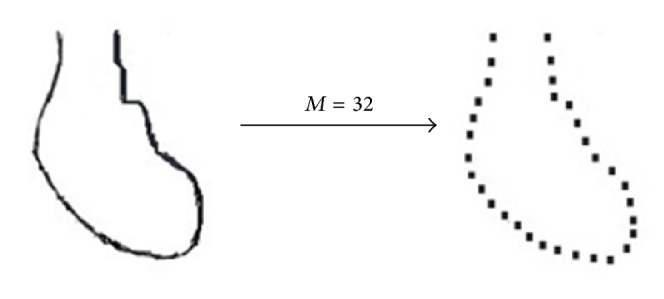
The result of the DR method for the heart 3D model.

Experiments of the heart 3D model set show that the effectiveness of using *M* = 16 is much lower than *M* = 32, whereas increasing the value of *M* to 64 does not lead to further improvements. Thus, in this experiment, we will use *M* = 32; the result of the DR method is as shown in Figure 6. Using 32 generated outline points calculates the distance from each point to the centroid (*x*_0_, *y*_0_) and then generates 32D feature vector *R* = (*r*_1_, *r*_2_,…, *r*_32_).

#### 3.1.3. The FVT Experiments

**Figure 7:**
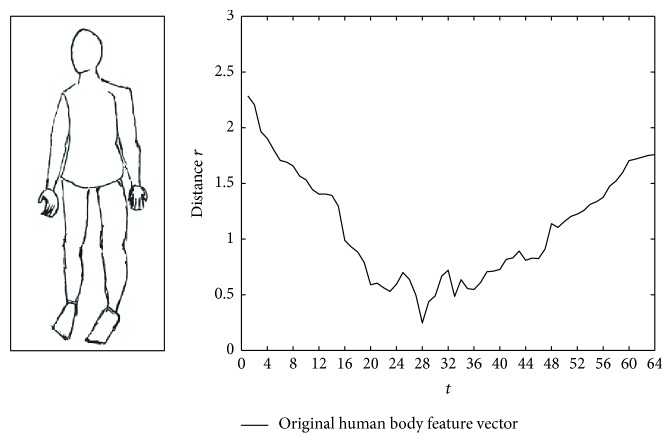
The original human body feature vector.

**Figure 8:**
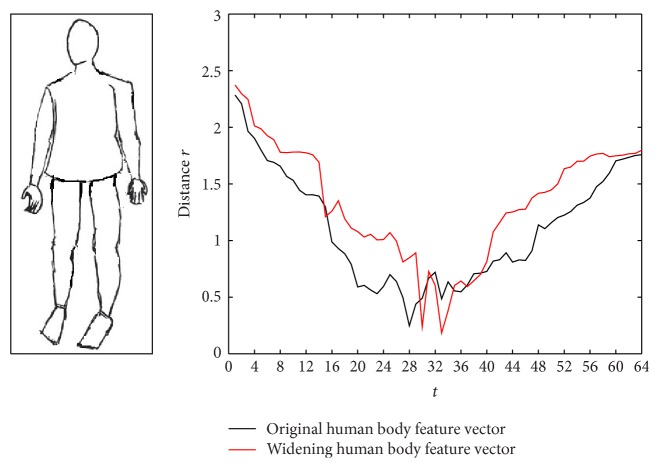
The widening human body feature vector.

**Figure 9:**
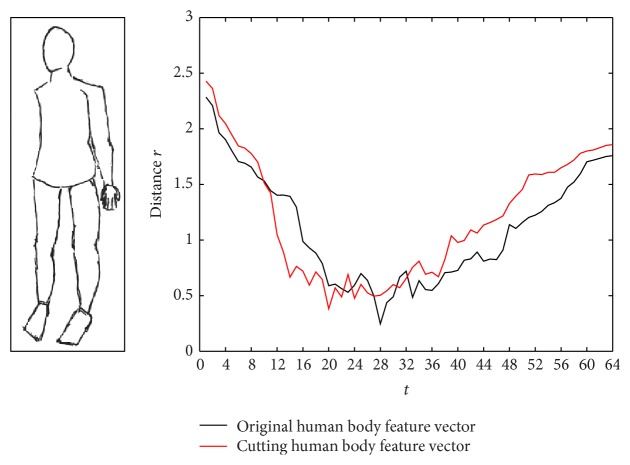
The cutting human body feature vector.

This paper considers the outline of the transformed human body, such as widening or cutting the human body. We select the *M* = 64 value using the DR method and calculate the distance from the outline point to the centroid $r_{t}=\sqrt{{\left(x_{t}-x_{0}\right)}^{2}+{\left(y_{t}-y_{0}\right)}^{2}}$. The feature vector *R* = (*r*_1_, *r*_2_,…, *r*_64_) is shown as follows.  Figure 7 shows the original human body feature vector, Figure 8 shows the widening human body feature vector, and Figure 9 shows the cutting human body feature vector.

Figures 8 and 9 show that the feature vectors have certain sensitivity to noise. Therefore we transform the existing feature vector *R* = (*r*_1_, *r*_2_,…, *r*_*N*_) and generate new feature vector *R*′ = (*r*_*k*_, *r*_*k*+1_,…, *r*_*N*_, *r*_1_,…, *r*_*k*−1_) to solve noise sensitive issue.

### 3.2. Algorithm Performance

For evaluation purposes, any 3D model in the same category of the query is considered relevant to that query, whereas all other models are considered irrelevant. To measure the retrieval effectiveness, we considered classical precision (*P*) and recall (*R*) metrics [36, 37] averaged over the set of processed queries. They are defined as follows:

(11) precision=relevant  correctly  retrievedall  retrieved,recall=relevant  correctly  retrievedall  relevant.

#### 3.2.1. Effect of the DR Method

**Figure 10:**
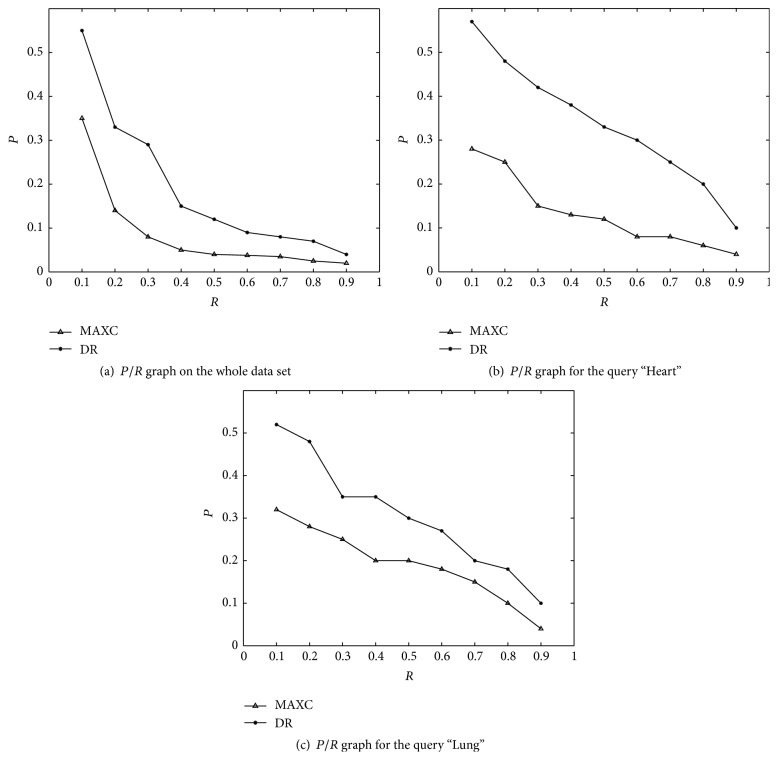
*P*/*R* comparing DR and MAXC method.

In our first experiment, we compare the DR method with the one based on the extraction from the boundary of the *M* points having the highest curvature values, hereafter called MAXC [38]. We do not consider methods that reduce dimensionality by simply resampling the original boundary every *N*/*M* points since this easily leads to missing significant shape details. Results in Figure 10(a) clearly show that DR method consistently outperforms MAXC in precision and that it is always positive even for higher recall levels. A similar trend can be also observed on specific query, as shown in Figures 10(b) and 10(c).

#### 3.2.2. Effect of the FVT Method

**Figure 11:**
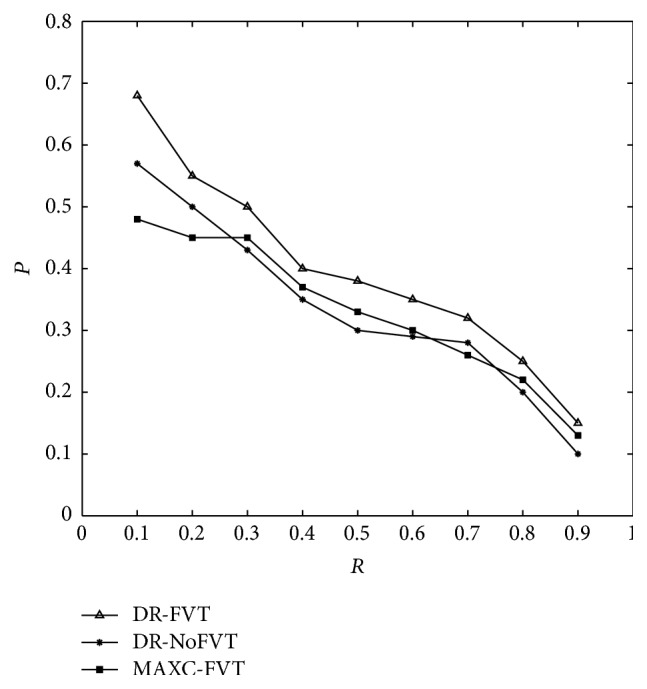
*P*/*R* graph for the query “Human Body.”

**Figure 12:**
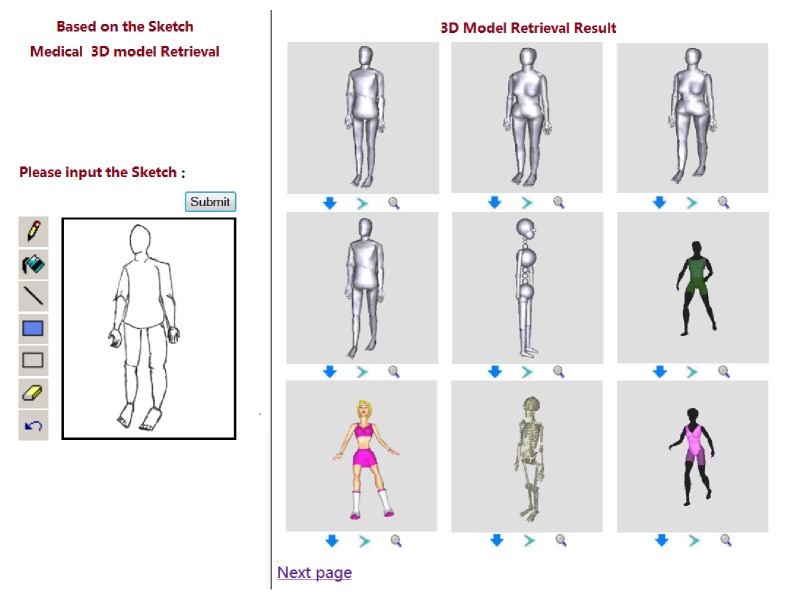
Results for the query “Human Body.”

In order to consider the effects of the FVT method, we compare the DRFVT method with the other two methods. One method uses the DR method and discards the FVT method, hereafter called DR-NoFVT. The other method uses the MAXC and the FVT method, called MAXC-FVT. For one specific query, Figure 11 shows the *P*/*R* graph for the query “Human Body” and Figure 12 for visualization of retrieval results. The experiment results demonstrate that the FVT method is very useful, and the DRFVT method gets better precision than other proposed methods.

## 4. Discussion and Conclusion

In this paper, we propose a novel medical freehand sketch 3D model retrieval method DRFVT which uses dimensionality reduction and feature vector transformation. It firstly provides a convenient interface to satisfy the user's design process and extract the outline of sketch based on eight-direction adaptive tracking algorithm. Secondly, the DR method reduces the dimensionality of feature vector; only the top *M* low frequency DFT coefficients are retained. These *M* coefficients are modified so as to achieve the invariance and then stored in the database. Finally, The FVT method is proposed, which generates a new feature vector to solve the problem of noise sensitivity. On this basis, the similarity between the sketch and the 3D model is calculated; thus the final retrieval results would be presented to the users. The experiment results demonstrate that our method achieves retrieval results more effectively and efficiently than other previously proposed methods.

The future of our work is to develop a user interaction feedback mechanism. After the users submit the query sketch, the system first provides a list of retrieved 3D models. Then, the users can refine the retrieval results by selecting the 3D models which they take as the good results. This feedback mechanism can not only provide more desirable retrieved 3D models to the user, but also enhance the user interaction just by making some easy choices. It would largely improve the effectiveness of our retrieval system [39, 40].

## Figures and Tables

**Table 1:** The normalized DFT coefficients.

Invariance	Modified coefficient
Translation	${\overset{⌢}{Z}}_{0}=0$
Scale	${\overset{⌢}{R}}_{m}=\frac{R_{m}}{R_{1}}$
Rotation	${\overset{⌢}{\theta }}_{m}=\theta_{m}-\frac{\theta_{-1}+\theta_{1}}{2}$
Starting point	${\overset{⌢}{\theta }}_{m}=\theta_{m}+m\frac{\theta_{-1}-\theta_{1}}{2}$

**Table 2:** Sample 3D models based on 6 categories.

3D model	Category	3D model	Category	3D model	Category
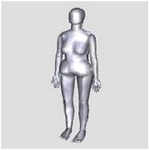	Body	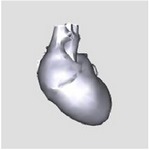	Heart	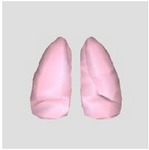	Lung

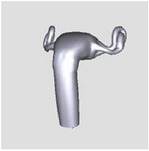	Ovary	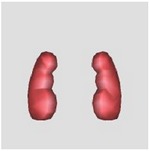	Kidney	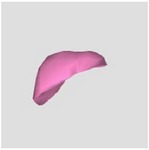	Liver
